# A Promising Breakthrough for Triple‐Negative Breast Cancer by Targeting the AKT and EZH2

**DOI:** 10.1002/mco2.70318

**Published:** 2025-08-05

**Authors:** Zhuoyun Liu, Yirong Li, Xinghua Long

**Affiliations:** ^1^ Department of Laboratory Medicine Zhongnan Hospital of Wuhan University Wuhan China

1

In a recent study published in *Nature*, Cichowski et al. [1] first revealed the effectiveness and potential mechanisms of combining AKT and EZH2 inhibitors (AKTi and EZH2i) for treating triple‐negative breast cancer (TNBC). The study elucidated how AKTi and EZH2i work synergistically to differentiate basal‐like TNBC cells into a more luminal state and hijack signaling pathways during normal breast degeneration to drive apoptosis in cancer cells, providing a new strategy for TNBC therapy.

The phosphoinositide 3‐kinase (PI3K)/AKT1 pathway, frequently hyperactive in breast cancer, is typically activated by PIK3CA mutations in hormone receptor (HR)‐positive luminal types. In contrast, phosphatase and tensin homolog (PTEN) loss predominantly drives the basal‐like TNBC subtype. Existing research indicates that while PI3Kα inhibitors benefit HR‐positive cancers, AKT inhibitors have shown limited efficacy in TNBC [2]. In the natural involution of the mammary gland, AKT promotes the restructuring and regression of breast tissue through the regulation of apoptosis. EZH2 is instrumental in maintaining the specific states of breast cells, thereby aiding their correct signaling responses during involution [3]. Cichowski's team hypothesizes that AKT inhibitors could render TNBC cells sensitive by promoting a luminal‐like phenotype. EZH2 is often overexpressed in breast cancer and plays an important role in maintaining the luminal progenitor state and limiting the differentiation of luminal cells in normal mammary epithelium of mice, thus being concerned by Cichowski et al.

In cell tests, the AKTi was added after TNBC cell lines had been pretreated for 5 days with the EZH2i. The results showed that 60% of the cell lines were susceptible to this combination treatment, significantly reducing cell numbers within 4 days. The AKT and EZH2 inhibitors displayed negligible cytotoxic effects when taken separately but showed strong and long‐lasting cytotoxic effects when taken together. Similar efficacy has been observed in multiple animal models, especially in patient‐derived xenograft (PDX) models, where significant tumor regression was only triggered when the drugs were combined. The findings underscore the therapeutic potential of this inhibitor pairing in TNBC, offering a promising treatment strategy. Furthermore, the minimal weight changes observed in mouse models posttreatment affirm the combination's favorable safety and tolerability profile.

The study leveraged RNA sequencing to delve into the impact of the EZH2/AKTi combination on TNBC cells, uncovering substantial alterations in gene expression posttreatment. Specifically, there was an upregulation of luminal‐associated genes such as GATA3 and ELF3, alongside a downregulation of basal and stem cell markers including KRT5, KRT14, and VIM. Complementary cyclic immunofluorescence imaging of HCI‐004 PDX tumors validated the in vivo transition of basal‐like TNBC cells toward a luminal phenotype. This cell state transition is an essential link in the therapeutic response, laying the stage for subsequent cell death induction.

Chikowski's team found that GATA3, as a key transcription factor, was significantly upregulated after the combined use of EZH2 and AKT inhibitors, which is crucial for driving basal‐like TNBC cells to adopt a luminal phenotype. They further elucidated that EZH2 inhibition enhances GATA3 expression by increasing chromatin accessibility in its enhancer regions. Small interfering RNA (siRNA) screening identified FOXO1 as a pivotal regulator influencing both treatment response and GATA3 expression. AKT inhibitors prevent FOXO1 phosphorylation, facilitating its binding to GATA3's enhancer and promoter, thereby promoting its expression. This coordinated control of FOXO1 activity by EZH2 and AKT inhibitors enhances GATA3 expression, fostering TNBC cell differentiation and tumor regression (Figure 1A).

**FIGURE 1:**
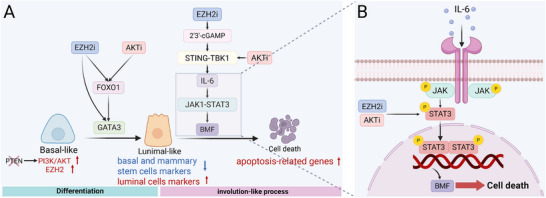
Synergistic inhibition of AKT and EZH2 in the treatment of triple‐negative breast cancer. (A) AKT and EZH2 inhibitors work synergistically to enhance FOXO1 expression, promoting its binding to the enhancer and promoter of GATA3, thereby facilitating GATA3 expression. EZH2i can increase chromatin accessibility in the GATA3 enhancer region, enhancing GATA3 expression. GATA3 drives basal‐like TNBC cells to differentiate into a luminal phenotype. Additionally, EZH2 inhibitors induce the formation of 2′3′‐cGAMP, while AKT inhibitors enhance the interaction between STING and TBK1. The combined effect promotes IL‐6 production, activating the JAK1–STAT3–BMF signaling pathway, which is essential for normal breast degeneration, leading to TNBC cell death. (B) IL‐6 binds to the cell membrane IL‐6 receptor, thereby triggering the JAK1–STAT3–BMF signaling cascade. Additionally, the synergistic effect of AKT and EZH2 inhibitors enhances STAT3 phosphorylation. Phosphorylated STAT3 (p‐STAT3) dimerizes and enters the nucleus, directly binding to the BMF promoter and driving its transcription, ultimately leading to TNBC cell apoptosis. P, phosphorylated (created with BioRender.com [https://biorender.com]).

Beyond inducing cell differentiation, the combined treatment can activate signaling pathways related to mammary gland involution to induce cell apoptosis. The study discovered that all susceptible cell lines exhibited a considerable upregulation of apoptosis‐related genes following combination therapy, particularly the proapoptotic protein BCL‐2‐modifying factor (BMF). siRNA‐mediated BMF knockdown rescued drug‐induced cytotoxicity, confirming its pivotal role. Interestingly, BMF plays an essential function in the mammary gland's natural involution and is regulated by the Janus Kinase 1 (JAK1)–signal transducer and activator of transcription 3 (STAT3) pathway. Therefore, the authors investigated the JAK1–STAT3 pathway and found that AKT and EZH2 inhibitors synergistically induce STAT3 phosphorylation. The authors additionally discovered that interleukin‐6 (IL‐6) is a crucial upstream regulator of this pathway. The EZH2 inhibitors can induce the formation of 2′3′‐cyclic GMP–AMP (cGAMP) by increasing reverse expression elements and endogenous retrovirus levels, while the AKT inhibitors can strengthen the interaction between stimulator of interferon genes (STING) and TANK‐binding kinase 1 (TBK1) (Figure 1A). The synergistic effect promotes the production of cytokine IL‐6, which binds to the IL‐6 receptor to activate the JAK1–STAT3–BMF pathway, stimulates the production of BMF, and then induces the death of TNBC cells (Figure 1B).

Cichowski's team found important differences in the epigenetic status of sensitive and resistant cells. Therefore, they utilized a machine learning approach to develop a classifier capable of predicting the sensitivity of TNBC cells to EZH2/AKT inhibitors, providing a potential tool for clinical screening of patients who may be sensitive to combination therapy. Meanwhile, Cichowski's team found that when used, GATA3 and STING agonists could reprogram drug‐resistant TNBC cells to restore sensitivity to EZH2/AKT inhibitors.

One of the main strengths of this study is that it discovers a previously unexplored pathway in breast cancer treatment—hijacking normal biological processes, such as involution, to combat cancer. The idea that mammary gland involution, which usually affects healthy tissue after lactation, can be used to eliminate cancer cells is a groundbreaking concept that adds new depth to understanding TNBC biology. Although the combination of AKT and EZH2 inhibition shows promise in preclinical models, further research is needed to evaluate its safety and potential off‐target effects in humans. In particular, since AKT and EZH2 play crucial roles in normal cell functions outside the tumor microenvironment, the long‐term effects and potential adverse reactions of inhibiting these pathways on patients must be carefully evaluated in clinical trials [4].

Intriguingly, both prostate cancer (PCa) and TNBC exploit lineage plasticity and epigenetic dysregulation to drive therapy resistance. In PCa, EZH2 silences differentiation genes (NKX3.1), inducing neuroendocrine differentiation and androgen receptor (AR) signaling loss, while PI3K/AKT hyperactivation (common in PTEN‐null tumors) suppresses AR via negative feedback [5]. AKT inhibition reactivates AR signaling in PTEN‐deficient PCa—a mechanism mirroring AKT/EZH2 roles in TNBC. We speculate that combined inhibition of EZH2 and AKT can reverse the neuroendocrine phenotype of PCa and synergistically enhance drug sensitivity of tumors. Similarly, histone deacetylases (HDACs)—epigenetic modulators like EZH2—silence tumor suppressors via chromatin remodeling. The development of HDAC/AKT dual‐target inhibitors holds promise for optimizing therapeutic efficacy in breast and PCas, offering a novel precision therapy option for patients harboring tumors characterized by PI3K/AKT pathway aberrations and epigenetic dysregulation.

In conclusion, Cichowski's work is a pioneering study in which the authors provide strong evidence for the synergistic effects of AKT and EZH2 inhibitors, valuable insights into the comprehensive analysis of the synergistic mechanism, and a new framework for the treatment of TNBC. Additionally, the sensitivity prediction model established through machine learning provides an essential theoretical basis for developing personalized treatment regimens. Although further clinical validation is needed, this study provides a new strategy for treating TNBC and a promising avenue for improving the prognosis of TNBC patients.

## Author Contributions

Z.L. drafted the manuscript and drew the figure. Y.L. reviewed the manuscript. X.L. drafted and reviewed the manuscript. All authors have read and approved the final manuscript.

## Conflicts of Interest

The authors declare no conflicts of interest.

## Ethics Statement

The authors have nothing to report.

## Data Availability

Not Applicable.
